# Exercise Training Could Improve Age-Related Changes in Cerebral Blood Flow and Capillary Vascularity through the Upregulation of VEGF and eNOS

**DOI:** 10.1155/2014/230791

**Published:** 2014-04-13

**Authors:** Sheepsumon Viboolvorakul, Suthiluk Patumraj

**Affiliations:** ^1^Inter-Department of Physiology, Graduate School, Chulalongkorn University, Bangkok 10330, Thailand; ^2^Center of Excellence for Microcirculation, Department of Physiology, Faculty of Medicine, Chulalongkorn University, Bangkok 10330, Thailand

## Abstract

This study aimed to investigate the effect of exercise training on age-induced microvascular alterations in the brain. Additionally, the association with the protein levels of vascular endothelial growth factor (VEGF) and endothelial nitric oxide synthase (eNOS) was also assessed. Male Wistar rats were divided into four groups: sedentary-young (SE-Young, *n* = 5), sedentary aged (SE-Aged, *n* = 8), immersed-aged (IM-Aged, *n* = 5), and exercise trained-aged (ET-Aged, 60 minutes/day and 5 days/week for 8 weeks, *n* = 8) rats. The MAPs of all aged groups, SE-Aged, IM-Aged, and ET-Aged, were significantly higher than that of the SE-Young group. The regional cerebral blood flow (rCBF) in the SE-Aged and IM-Aged was significantly decreased as compared to SE-Young groups. However, rCBF of ET-Aged group was significantly higher than that in the IM-Aged group (*P* < 0.05). Moreover, the percentage of capillary vascularity (%CV) and the levels of VEGF and eNOS in the ET-Aged group were significantly increased compared to the IM-Aged group (*P* < 0.05). These results imply that exercise training could improve age-induced microvascular changes and hypoperfusion closely associated with the upregulation of VEGF and eNOS.

## 1. Introduction

According to a United Nations report [1], the worldwide elderly population is growing rapidly. Since 1950, the proportion of older persons has been rising steadily, growing from 8% in 1950 to 11% in 2007, and is expected to reach 22% in 2050. The rapid growth of the global aging population has profound implications for many aspects of human health. In particular, the aging process results in a decline in body functions, and the vascular system is no exception. Angiogenesis, the development of new microvessels from preexisting vasculature, is delayed and altered with age [2]. The subsequent impairment of angiogenesis is detrimental to both the revascularization of ischemic organs and the repair of injured tissues.

Oxidative stress in a physiological setting can be defined as an excessive bioavailability of reactive oxygen species (ROS), which is the net result of an imbalance between the production and destruction of ROS (with the latter being influenced by antioxidant defenses). The “oxidative stress theory” of aging is a prevalent theory that proposes that a progressive and irreversible accumulation of oxidative damage caused by ROS impacts critical aspects of the aging process and contributes to impaired physiological function, an increased incidence of disease and a reduction in life span [3]. ROS are the primary causal factor underlying aging-associated declines in physiological function [3]. In addition, it has been demonstrated that the pathophysiology of impaired angiogenesis may also be related to the production of ROS [4].

Several studies have shown that exercise prevents vascular dysfunction, which is associated with a reduction in oxidative stress [5, 6]. In aging humans, it has been demonstrated that regular aerobic exercise improves the regional cerebral blood flow (rCBF) in various relevant brain structures in response to cognitive tasks along with better task performance [7]. Endurance exercise training also represents a beneficial tool for the stimulation of vascular angiogenesis in various organs [8–10]. The previous studies showed that 30 minutes of treadmill exercise each day for 3 weeks could be able to increase angiogenic factor, VEGF, within the cerebral vasculature of rats [9].

Angiogenesis induced by exercise has been reported for its association with an increase in the expression of angiogenic factors. Lloyd et al. [11] reported that exercise training induces angiogenesis due to the activation of angiopoietin and vascular endothelial growth factor (VEGF). Iemitsu et al. [12] reported that swim training ameliorates the aging-induced reduction in capillary density and demonstrated a related decrease in the expression of VEGF and its receptors Flt-1 and Flk-1. Moreover, it has been reported that exercise-induced shear stress could increase angiogenesis via NO-dependent manner [13, 14]. However, the link among the roles of exercise training in shear-stress induced eNOS, the upregulation of VEGF with FLK-1 expression, and oxidative stress have not been clarified in age-induced brain microvascular changes. Therefore, the present study aimed to investigate the effect of exercise training on age-induced brain microvascular alterations, with possible underlying mechanisms involving the regulation of VEGF and endothelial nitric oxide synthase (eNOS).

## 2. Materials and Methods

### 2.1. Animal Preparation

Male Wistar rats were divided into four groups: sedentary-young (aged 4–6 months) (SE-Young, *n* = 5), sedentary-aged (aged 23-24 months) (SE-Aged, *n* = 8), immersed-aged (IM-Aged, *n* = 5), and exercise trained-aged (ET-Aged, *n* = 8) rats. This study was approved by the Ethics Committee on the Care and Use of Laboratory Animals of the Faculty of Medicine Chulalongkorn University. The present study was conducted in accordance with the guidelines for laboratory animals established by the National Research Council of Thailand (1999).

In this experiment, the age-induced microvascular alterations will be demonstrated by the statistical analysis between sedentary-young (aged 4–6 months) (SE-Young, *n* = 5) and sedentary-aged (aged 23-24 months). These sedentary rats were subjected to the same swimming-room environment as the exercise trained-aged animals, except they remained in their cages.

The effects of exercise training in aging group will be a statistical comparison between immersed-aged (IM-Aged, placed in the swimming tanks with 5 cm water depth for 30 minutes/day and 5 days/week for 8 weeks, *n* = 5) and the exercise trained-aged (ET-Aged, 60 minutes/day and 5 days/week for 8 weeks, *n* = 8) rats. To minimize the possible stress effects associated with cold or hot water exposure, therefore, the IM-aged group was performed according to the modified methods of Iemitsu et al. and Eksakulkla et al. [15, 16].

### 2.2. Exercise Training Program

The swimming exercise protocol involved nonimpact endurance exercise with moderate intensity and was modified from the methods of Iemitsu et al. and also from Eksakulkla et al. [15, 16]. Each day, the animals were transported to an exercise training room and swam individually in cylindrical tanks with a diameter and height of 50 and 65 cm, respectively, with water at a depth of 50–55 cm. The rats were exercised once per day between 2:00 and 4:00 p.m. for 5 days/week. The animals swam for 15 minutes/day for the first 2 days, and the swimming time was then gradually increased each week from 15 to 60 minutes/day. Thereafter, the trained-aged group continued to swim for 7 weeks. Thus, the trained-aged group received 8 weeks of swim training. To minimize the stress associated with exposure to hot or cold water, the water temperature was kept at 33–36°C. At the end of each training session, the rats were dried with a towel and hair dryer. The sedentary-young and sedentary-aged animals were transported to the same training room but remained in their cages during the training hour and were handled daily. The immersed-aged animals were placed individually in cylindrical tanks filled with water to a depth of 5 cm; this water was controlled to be the same temperature as that used for the trained-aged animals. The immersed-aged rats were placed in the swimming tanks for 30 minutes/day and 5 days/week for 8 weeks. After 8 weeks of swim training, the immersed-aged and trained-aged animals were made to rest for at least 24 hours before they were subjected to the experiment.

### 2.3. Cerebral Microvascular Imaging

On the day of the experiment, the body weights of the rats, which were fasted overnight, were measured, and the rats were then anesthetized with pentobarbital sodium (60 mg/kg body weight, intraperitoneally). Their body temperature was maintained at 37°C with a homoeothermic blanket. The rats were tracheotomies and mechanically ventilated with room air containing supplemental oxygen using an animal ventilator (Kent Scientific, USA). A catheter was placed into the right femoral artery to obtain a blood sample for measurement of blood gases with a portable blood gas analyzer (iSTAT Abbott Point of Care Inc., USA). The arterial blood gases were measured before and after the experiment, and the blood gas values were recorded and remained stable within normal ranges (pH: 7.35–7.45; PO_2_: 80–120 mmHg; PCO_2_: 35–45 mmHg). Another catheter was placed in the left femoral vein for the injection of a fluorescent tracer, supplemental anesthesia, and the replacement of fluids.

Each rat was placed in a homemade stereotaxic apparatus. The animal's head was fixed in the stereotaxic frame, and the skull was exposed via a midline incision. Using a dissecting microscope (Olympus, Tokyo) and a low-speed drill (World Precision Instrument Inc., USA), a circular cranial window of 3 mm in diameter was created over the left parietal cortex (2 mm posterior to the left coronal suture and 2 mm lateral to the linear temporalis). To prevent the overheating of the cerebral cortex during drilling, the skull was cooled by the periodic application of room temperature saline. The dura mater was carefully removed using a pair of microscissors (PMS GmbH, Germany). A custom-made stainless metal ring (8 mm in diameter) was then sealed over the cranial window using dental cement (GC Dental Products Corp., Japan). The cerebral cortex was suffused with artificial cerebral spinal fluid (118 mM NaCl, 4 mM KCl, 1.2 mM NaH_2_PO_4_, 5 mM D-glucose, 1.5 mM CaCl_2_, 1.2 mM MgSO_4_, and 25 mM NaHCO_3_).

**Figure 1:**
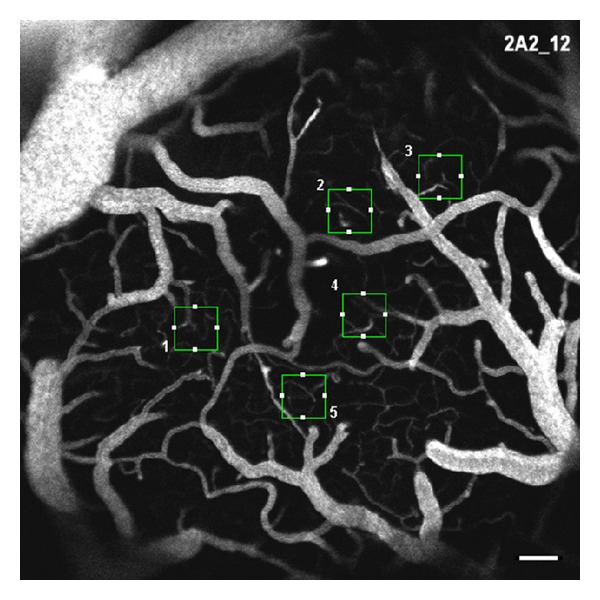
Representative grey-scale fluorescent image of the microvasculature in the cortical surface. The five rectangular boxes demarcate the microvessels (diameter < 10 *μ*m). Scale bar = 100 *μ*m.

After the surgical exposure of the cerebral cortex, measurements of the regional cerebral blood flow were performed using a laser Doppler perfusion monitoring unit (PeriFlux System 5000, Perimed AB, Sweden). The arterial blood gases were sampled before and after the direct imaging of the cerebral microvascular network. To visualize the microvessels, 0.2 mL fluorescein isothiocyanate-dextran (FITC) (50 mg/mL; molecular weight 250 kDa) was injected intravenously. The microvascular network was imaged using a laser scanning confocal microscopy system (Eclipse C1 Plus, Nikon, Japan). This system included an upright fluorescence microscope equipped with an epi-illuminator for fluorescence, which was provided by a high-pressure mercury lamp (Ushio Inc., Japan) using an appropriate filter for FITC. The light source for the confocal microscope was an argon-ion laser whose wavelength was 488 nm. The cranial window was explored with a 10x objective lens (Plan Fluor 10x dry, Nikon, Japan). The cerebrovascular network was recorded using a computer-based frame grabber (EZ-C1, Nikon, Japan) with a controlled gain, offset, and exposure time. The microvascular network was visualized and recorded within 5 minutes (shown in Figure 1). The collected fluorescent images were analyzed offline for capillary vascularity using GLOBAL Lab Image/2 software (Data Translation, Inc., USA).

After imaging, a cannula was inserted into the apex of the left ventricle to allow for the perfusion of the brain with 200 mL ice-cold phosphate buffer saline (PBS) containing heparin (25 UI/mL). The brain was then removed and stored at −80°C until the cerebral microvessels were subsequently isolated. The isolated brain microvessels were used for the determination of the VEGF, Flk-1, and eNOS levels by an immunoassay technique.

### 2.4. Measurement of the Regional Cerebral Blood Flow

The regional cerebral blood flow (rCBF) was measured by laser Doppler perfusion monitoring with a laser Doppler probe (PeriFlux System 5000, Perimed AB, Sweden) interfaced to a laptop equipped with the PeriSoft data-acquisition software (Perimed Systems, Inc., Sweden). Laser Doppler flowmetry is a noninvasive method of determining tissue perfusion and is becoming a widely used technique. Its underlying principle is that the laser light backscattered from the tissue is spectrally broadened by the Doppler shifts produced by moving red blood cells. For each measurement, the laser Doppler probe was placed perpendicular to the cortical surface, and placement over any areas with large vessels was avoided. The rCBF data for each animal was obtained from three cortical regions and is expressed in arbitrary “perfusion units.”

### 2.5. Determination of Capillary Vascularity

**Figure 2:**
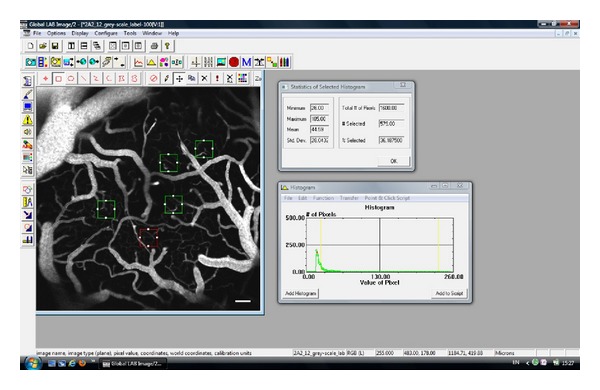
GLOBAL Lab Image/2 software showing the capillary vascularity (CV) of the ROI within the red rectangle. Here, the total number of pixels within the ROI was 1,600.00, and the number of pixels within the capillaries was 579.00. The CV was therefore estimated to be 36.19%.

The recorded fluorescent images were analyzed for capillary vascularity using GLOBAL Lab Image/2 software (Data Translation, Inc., USA) [10]. All images were acquired using the same camera settings (gain, offset, and exposure time). The RGB images were converted into binary images, in which the vascular pixels and perivascular pixels were discriminable based on their gray-scale intensities. The capillary vascularity was analyzed as described in detail in the previous study [10] using a 100 × 100 *μ*m rectangular region of interest (ROI). Five ROI were selected for each image, and each ROI was oriented to include microvessels (<10 *μ*m in diameter) (Figure 2).

The software calculated the percentage of capillary vascularity using the following equation:

(1) Capillary  vascularity  (%) =Number  of  pixels  located  within  the  vessels  of  each  ROITotal  number  of  pixels  in  each  ROI   ×100.

The determination of capillary vascularity was performed using 5 ROIs/image, 3 images/animal, and 5–8 animals/group.

### 2.6. Isolation of Brain Microvessels

Brain microvessels were isolated according to the method of Yamakawa et al. [17]. The brain was freed of the cerebellum and brain stem and then rinsed in ice-cold PBS. The chopped tissue was homogenized in 3 mL of ice-cold isotonic sucrose buffer (0.32 M sucrose, 3 mM HEPES [pH 7.4]) using a Potter-Elvehjem glass-Teflon homogenizer (Glas-Col, USA) with 10 strokes at 3,000 rpm and 4°C. The homogenate was centrifuged at 1,000 ×g for 10 minutes at 4°C. The supernatant was discarded, and the pellet was resuspended in 3 mL of ice-cold sucrose buffer, followed by centrifugation at 1,000 ×g for 10 minutes at 4°C. The pellet was resuspended in ice-cold sucrose buffer and centrifuged twice at 100 ×g for 10 minutes at 4°C. The supernatants of the final two centrifugations were pooled and centrifuged at 200 ×g for 2 minutes at 4°C. The pellet was washed twice with ice-cold sucrose buffer and once with ice-cold PBS + 0.1% bovine serum albumin (BSA) at 200 ×g for 2 minutes at 4°C for each step. The pellet was resuspended in 1 mL of ice-cold PBS + 0.1% BSA, and the purity of the microvessel preparation was evaluated. The suspension was centrifuged at 14,000 ×g for 2 minutes at 4°C. The final pellet was stored at −80°C.

### 2.7. Determination of Malondialdehyde (MDA) Levels

At the end of the experiments, whole blood was collected from the abdominal aorta for the determination of the malondialdehyde (MDA) level (a common oxidative stress marker) using a commercial assay kit (Cayman Chemical, MI).

### 2.8. Immunoassay for VEGF, Flk-1, and eNOS

Isolated brain microvessels were homogenized in 500 *μ*L of ice-cold RIPA buffer containing a protease inhibitor cocktail (Sigma, USA). The homogenate was centrifuged at 1,000 ×g for 5 minutes at 4°C. The supernatant, as the postnuclear fraction, was aliquot and stored at −80°C. The protein concentration of the postnuclear supernatant was determined using a bicinchoninic acid (BCA) assay kit (Pierce, USA). The supernatant aliquots were used for the quantification of the VEGF, Flk-1, and eNOS protein levels with commercial immunoassay kits (R&D Systems, USA).

The VEGF levels were quantified using a sandwich enzyme immunoassay technique and an ELISA kit (MMV00, R&D Systems, USA). The standard solution or the samples were added to a 96-well plate that was precoated with a polyclonal antibody specific for VEGF. The samples were incubated for 2 hours, and after washing the plate, a substrate solution was added to the wells. The enzyme reaction yielded a blue product that turned yellow when the stop solution was added. The intensity of the color was proportional to the amount of VEGF bound in the initial step. The sample values were then compared to the standard curve.

Flk-1 expression was measured using a solid-phase sandwich ELISA kit (MVR200B, R&D Systems, USA). The standards and samples were pipetted into the wells, and any Flk-1 present was bound by the immobilized antibody. After washing away any unbound substances, an enzyme-linked polyclonal antibody specific for Flk-1 was added to the wells. Following a wash to remove any unbound antibody-enzyme reagent, a substrate solution was added to the wells. The enzyme reaction yielded a blue product that turned yellow when the stop solution was added. The intensity of the color measured was proportional to the amount of Flk-1 bound in the initial step. The sample values were then compared to the standard curve.

The eNOS protein levels were quantitated using a commercially available solid-phase sandwich ELISA kit (DEN00, R&D Systems, USA) following the manufacturer's instructions. This assay employed a quantitative enzyme immunoassay technique in which a monoclonal antibody specific for eNOS was precoated onto a microplate. The standards and samples were pipetted into the wells, and any eNOS present was bound by the immobilized antibody. After washing away any unbound substances, an enzyme-linked polyclonal antibody specific for eNOS was added to the wells. Following a wash to remove any unbound antibody-enzyme reagent, a substrate solution was added to the wells, and color developed in proportion to the amount of eNOS bound in the initial step. The color development was stopped after 10° min, and the intensity of the color was measured. The eNOS concentration of each sample was then calculated from the standard curve.

### 2.9. Statistical Analysis

The results are expressed as the mean ± standard error of mean (SEM). Any significant differences between groups were determined using the one-way analysis of variance (one-way ANOVA), and differences between pairs of mean values were evaluated by the least significant difference (LSD) test. To evaluate the difference in between the immersed-aged and exercise trained-aged groups, Student's* t*-test for unpaired values was used. Differences were statistically significant if the statistical probability (*P* value) was less than 0.05. The data were analyzed using SPSS 16.0 for Windows (SPSS Inc., USA).

## 3. Results

### 3.1. Biochemical Parameters and Physiological Characteristics

The body weights and mean arterial blood pressures (MAPs) of the rats in the sedentary-young, sedentary-aged, immersed-aged, and trained-aged groups are summarized in Table 1. It was found that both the SE-Aged and IM-Aged rats had significantly higher body weights compared to the SE-Young group. In addition the data also showed a significant decline in the body mass of the ET-Aged group (591.50 ± 14.68 g) when compared to IM-Aged group (686.80 ± 16.87 g) (*P* < 0.05).

The MAPs of the SE-Aged (130.00 ± 5.00 mmHg), IM-Aged (130.39 ± 2.69 mmHg), and ET-Aged (115.79 ± 4.30 mmHg) groups were significantly higher than that of the SE-Young group (99.11 ± 6.04 mmHg, *P* < 0.05). However, the ET-Aged group had a significantly reduced MAP compared to the IM-Aged group (*P* < 0.05).

### 3.2. Effect of Exercise Training on Regional Cerebral Blood Flow and Brain Capillary Vascularity

During the experiment, the physical condition of the rats was broadly unchanged. The blood gas levels before and after the experiments were monitored. None of the differences observed in the blood gas values before and after the experiment were statistically significant.

**Figure 3:**
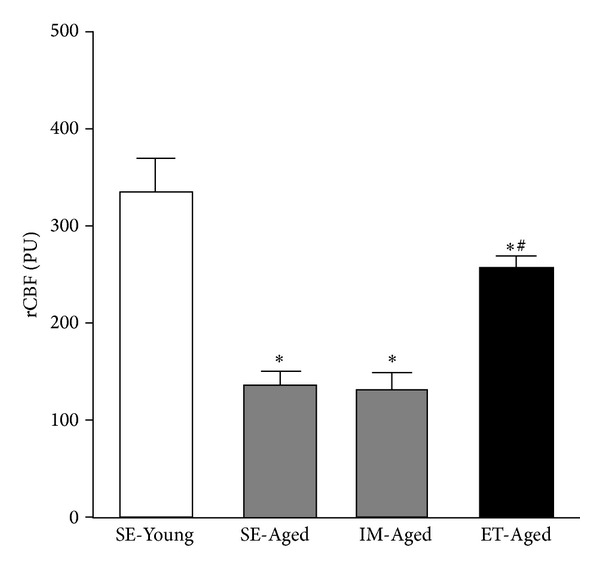
Effect of exercise training on regional cerebral blood flow (rCBF) in sedentary-young (SE-Young), sedentary-aged (SE-Aged), immersed-aged (IM-Aged), and trained-aged (ET-Aged) rats. *P < 0.05, significantly different from the SE-Young group. ^#^ *P* < 0.05, significantly different from the IM-Aged group.

**Figure 4:**
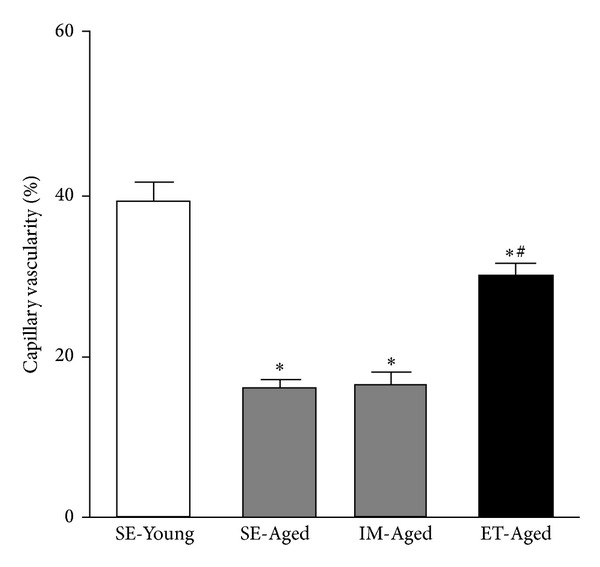
The capillary vascularity of the SE-Aged (15.85 ± 1.25%), IM-Aged (16.46 ± 1.59%), and ET-Aged (29.81 ± 1.64%) groups was significantly reduced compared with the SE-Young group (39.25 ± 2.18%). The capillary vascularity of the ET-Aged group was significantly greater than that of the SE-Aged group. *P < 0.05, significantly different from the SE-Young group. ^#^ *P* < 0.05, significantly different from the IM-Aged group.

The rCBF and capillary vascularity of the SE-Young, SE-Aged, IM-Aged, and ET-Aged groups are summarized in Figures 3 and 4.

The rCBF was significantly diminished (*P* < 0.05) in the SE-Aged (134.99 ± 14.74 PU), IM-Aged (131.09 ± 17.53 PU), and ET-Aged (255.83 ± 13.17 PU) groups compared to the SE-Young group (334.27 ± 35.00 PU) (Figure 3). However, the rCBF in the ET-Aged group was significantly higher than that in the IM-Aged group (*P* < 0.05).

The effect of exercise training on brain capillary vascularity in aging rats was illustrated in Figure 4. The capillary vascularity of the SE-Aged (15.85 ± 1.25%), IM-Aged (16.46 ± 1.59%), and ET-Aged (29.81 ± 1.64%) groups was significantly reduced (*P* < 0.05) compared with the SE-Young group (39.25 ± 2.18%). The capillary vascularity of the ET-Aged group (29.81 ± 1.64%) was significantly greater than that of the IM-Aged group (16.46 ± 1.59%) (*P* < 0.05).

**Figure 5:**
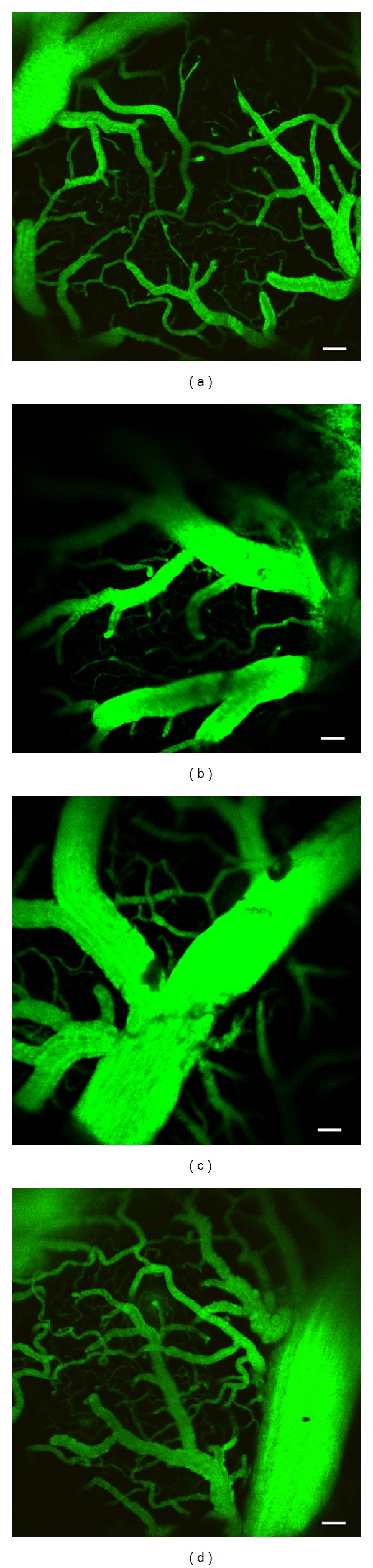
Representative images of the microvascular networks of sedentary-young (a), sedentary-aged (b), immersed-aged (c), and trained-aged (d) rats. The networks were visualized via a cranial window with a laser-scanning fluorescence confocal microscope with 10x objective lens using FITC-dextran as a fluorescent tracer. Scale bar = 100 *μ*m.

Images of the microvascular networks for the SE-Young, SE-Aged, IM-Aged, and ET-Aged groups were visualized by FITC-dextran under a laser-scanning fluorescence confocal microscope with a 10x objective lens (Figure 5). In the SE-Young group, the microvascular network exhibited a rich network of capillaries with a structurally intact architecture (Figure 5(a)). In contrast, the capillary network in both the SE-Aged and IM-Aged rats showed marked alterations, with a reduced capillary density and striking abnormalities in shape (Figures 5(b) and 5(c)) compared to the SE-Young rats. However, in the ET-Aged rats, the capillaries appeared to have developed a greater density and size (Figure 5(d)) as a result of exercise training.

### 3.3. VEGF, Flk-1, and eNOS

**Figure 6:**
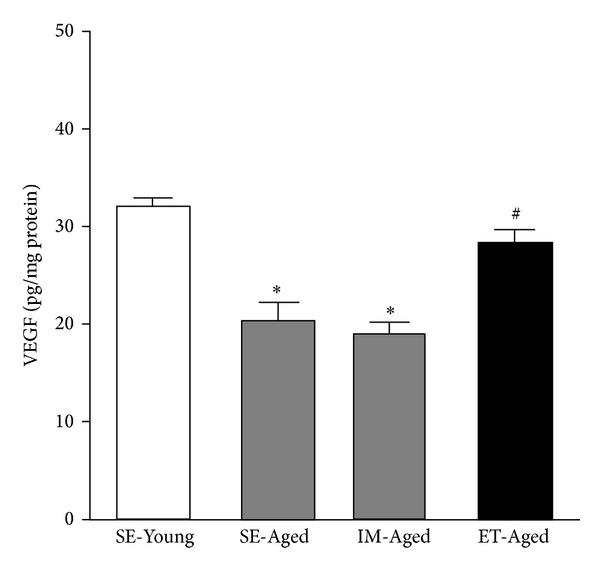
Effect of exercise training on the level of vascular endothelial growth factor (VEGF) in the brain microvessels of sedentary-young (SE-Young), sedentary-aged (SE-Aged), immersed-aged (IM-Aged), and trained-aged (ET-Aged) rats. The VEGF level was significantly lower (*P* < 0.05) in the SE-Aged (20.51 ± 1.75 pg/mg protein) and IM-Aged (19.07 ± 1.27 pg/mg protein) groups compared with the SE-Young group (32.27 ± 0.93 pg/mg protein). The level of VEGF in the ET-Aged (28.35 ± 1.53 pg/mg protein) group was significantly increased when compared with the IM-Aged group (19.07 ± 1.27 pg/mg protein). *P < 0.05, significantly different from the SE-Young group. ^#^ *P* < 0.05, significantly different from the IM-Aged group.

The VEGF level was significantly lower (*P* < 0.05) in the SE-Aged (20.51 ± 1.75 pg/mg protein) and IM-Aged (19.07 ± 1.27 pg/mg protein) groups compared with the SE-Young group (32.27 ± 0.93 pg/mg protein) (Figure 6). Swim training resulted in a significant increase (*P* < 0.05) in the level of VEGF in the ET-Aged (28.35 ± 1.53 pg/mg protein) group when compared with the IM-Aged group (19.07 ± 1.27 pg/mg protein). Additionally, there was no significant difference in the VEGF levels between the SE-Young and ET-Aged groups (*P* = 0.075).

**Figure 7:**
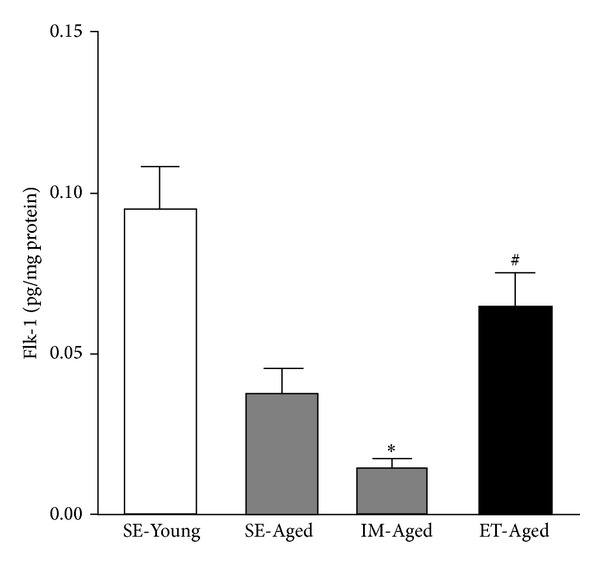
Effect of exercise training on the level of vascular endothelial growth factor receptor 2 (Flk-1) in the brain microvessels of sedentary-young (SE-Young), sedentary-aged (SE-Aged), immersed aged (IM-Aged), and train-aged (ET-Aged) rats. *P < 0.05, significantly different from the SE-Young group. ^#^ *P* < 0.05, significantly different from the IM-Aged group.

The level of Flk-1 in the IM-Aged group (0.014 ± 0.003 pg/mg protein) was significantly lower (*P* < 0.05) than that in the SE-Young group (0.076 ± 0.021 pg/mg protein) (Figure 7). Although the level of Flk-1 in the SE-Aged group (0.038 ± 0.008 pg/mg protein) was lower than that in the SE-Young group; this difference was not statistically significant (unpaired* t*-test, *P* = 0.05). However, the level of Flk-1 in the ET-Aged group (0.064 ± 0.011 pg/mg protein) was showing more significant increase than that in the IM-Aged group (0.014 ± 0.003 pg/mg protein) (unpaired* t*-test, *P* < 0.05).

**Figure 8:**
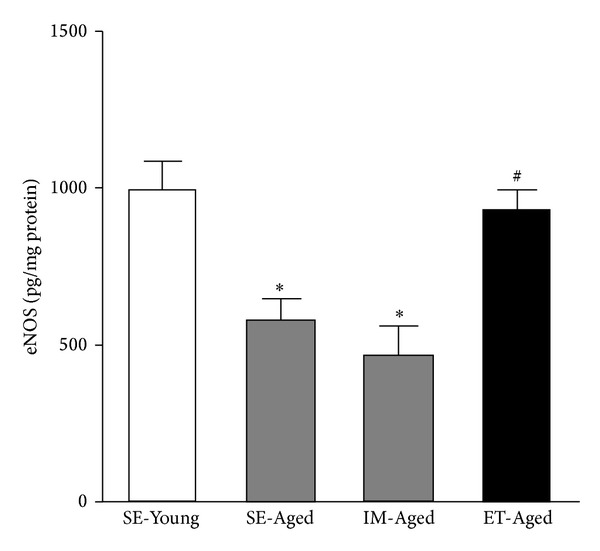
Effect of exercise training on the level of endothelial nitric oxide synthase (eNOS) in the brain microvessels of sedentary-young (SE-Young), sedentary-aged (SE-Aged), immersed-aged (IM-Aged), and trained-aged (ET-Aged) rats. Both the SE-Aged and IM-Aged groups had significantly lower eNOS levels (575.62 ± 70.14 and 459.94 ± 98.02 pg/mg protein, resp.) compared to the SE-Young group (994.39 ± 88.49 pg/mg protein). The eNOS level in the ET-Aged group (926.75 ± 65.08 pg/mg protein) was significantly increased when compared with the IM-Aged group (459.94 ± 98.02 pg/mg protein). *P < 0.05, significantly different from the SE-Young group. ^#^ *P* < 0.05, significantly different from the IM-Aged group.

Both the SE-Aged and IM-Aged groups had significantly lower (*P* < 0.05) eNOS levels (575.62 ± 70.14 and 459.94 ± 98.02 pg/mg protein, resp.) compared to the SE-Young group (994.39 ± 88.49 pg/mg protein) (Figure 8). Regular swimming resulted in a significantly elevated (*P* < 0.05) eNOS level in the ET-Aged group (926.75 ± 65.08 pg/mg protein) compared with the IM-Aged group (459.94 ± 98.02 pg/mg protein). However, the level of eNOS in the ET-Aged group was not significantly different (*P* = 0.5480) from that in the SE-Young group.

**Figure 9:**
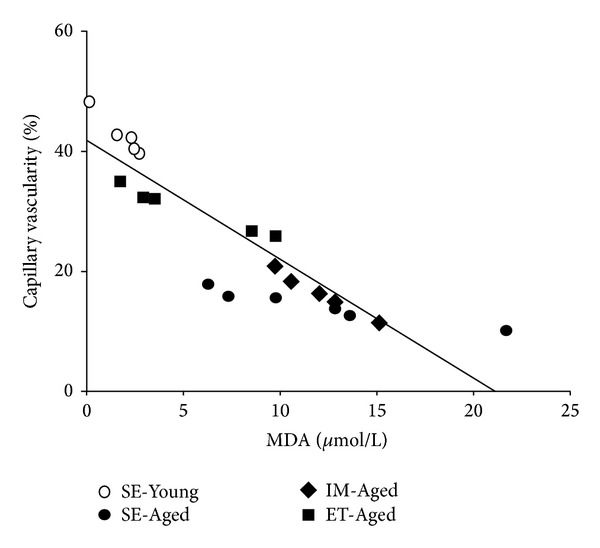
Relationship between the MDA level (*x*) and brain capillary vascularity (*y*) in sedentary-young (SE-Young), sedentary-aged (SE-Aged), immersed-aged (IM-Aged), and trained-aged (ET-Aged) rats.

The data on the capillary vascularity and MDA level in the SE-Young, SE-Aged, IM-Aged, and ET-Aged groups were collected and plotted in Figure 9. A linear relationship existed between the capillary vascularity and MDA level with a correlation coefficient of *R* ^2^ = 0.7958. The linear regression equation was expressed as *y* = −1.9368*x* + 40.999.

## 4. Discussion

In the present* in vivo* animal study, the protective effects of moderate exercise training on brain microvessels against age-induced microvascular changes were revealed. The deterioration of capillary vascularity and regional blood perfusion was found in the aging brain. Moreover, these results indicated that a moderate exercise training program could attenuate the decreases in brain capillary vascularity and blood perfusion and could significantly reduce age-induced oxidative stress. The upregulation of VEGF and eNOS, which are two key angiogenic proteins, may play a role in the protective effect of exercise training in the amelioration of brain microvascular perfusion during aging.

### 4.1. Effects of Exercise Training on Physiological Adaptations during Aging

The present study demonstrated the alterations in physiological characteristics induced by aging, including changes in body weight, MAP, and oxidative stress (Table 1). The greater body weight observed in the old rats occurred due to alterations in their body fat content and distribution and a reduction in their skeletal muscle mass and strength, which are common physiological changes found in the elderly [18]. It is well established that exercise training can reduce body mass in old age, which is primarily caused by a reduction in fat mass, as the capacity for fat oxidation is enhanced by endurance exercise [19]. In the current study, we also observed a significant decline in the body mass of the trained-aged group as a result of the swim training program.

The present data demonstrated that hypertension occurred in the SE-Aged and IM-Aged groups. Multiple mechanisms have been reported for the pathogenesis of hypertension in the elderly, including endothelial dysfunction (loss of vasomotor regulation), the elevation of oxidative stress, inflammation, cellular apoptosis, and increased concentrations of active metabolites (increased myogenic constriction) [20]. Interestingly, in the present study, the MAP was significantly lower in the ET-Aged rats compared to both the SE-Aged and IM-Aged groups. This finding suggests that age-induced hypertension can be ameliorated by the regular moderate exercise training program used in this study. A large body of evidence affirms that aerobic endurance training can lower high blood pressure, at least in part, by abolishing the imbalance between endothelium-derived relaxing and constricting factors [15, 21–23].

The present data demonstrated that the plasma MDA levels were significantly higher in both the IM-Aged and SE-Aged groups compared to the SE-Young group. In addition, swim training of moderate intensity also produced a lower plasma MDA level in the ET-Aged group compared to the SE-Aged group. Based on the free radical theory of aging, various oxygen species oxidize lipids, resulting in a diverse array of peroxide products [24]. Although a single bout of exercise in aging has been shown to enhance the level of ROS, the regular exercise training results in lower levels of lipid peroxidation [25]. Endurance exercise is well known to enhance the capacity for antioxidant defense, including an enhanced activity of free radical scavenging enzymes (e.g., SOD, glutathione peroxidase, and catalase) and mitochondrial oxidative enzymes (e.g., citrate synthase and NADH oxidase), in both young and old rats [26, 27].

The present study demonstrates that an 8-week swim training protocol results in a reduction of body weight and resting MAP as well as attenuated oxidative stress in aging rat model. We therefore conclude that adequate endurance training can be achieved using our swimming exercise program.

### 4.2. Effect of Exercise Training on Microvascular Changes in Aging Rat Brain

The present study describes our direct* in situ* investigation of cerebral microcirculation through the cranial window. The current method is based on the combined use of fluorescent intravascular tracers and laser-scanning confocal fluorescence microscopy and was developed for the real-time* in vivo* study of cerebral microcirculation in rats. The main advantage of laser-scanning confocal fluorescence microscopy is that it makes possible the high-quality visualization of the microvascular network from the surface through to the intraparenchyma at a depth of 100–200 *μ*m. For short periods of* in situ* exploration, the brain tissue was not damaged by the laser illumination, and the normal physiological status was maintained (stable arterial blood gas).

A reduction in cerebral blood flow has been found to occur with advancing age. The present study confirmed this phenomenon by demonstrating a markedly lower regional cerebral blood flow in both the SE-Aged and IM-Aged groups (Figure 3). The impaired vasodilation response of the cerebral arteriole during aging [27] may lead to cerebral hypoperfusion, which is related to the depletion of the cerebrovascular reserve, leading to an increased susceptibility of the brain to vascular insufficiency [28]. The declining rCBF and energy metabolism of the aging brain appear to have well-described morphological correlates. At the level of the cerebral microvessels, both the capillary density of distinct brain regions and the ultrastructure of the capillary walls are prone to age-related alterations [29]. Several groups have reported this reduction in cerebral capillary density in both humans [30, 31] and experimental animals [32–34]. The current data also demonstrated that the aging animal groups had a significantly lower brain capillary vascularity, as measured by an index of capillary density, compared with the young group (Figure 4).

Age-related brain microvascular rarefaction has the potential to result in inadequate blood flow to the brain. The mechanism for age-related changes in the brain microvasculature appears to correlate, at least in part, with a decline in growth factors that mediate angiogenesis. Vascular endothelial growth factor (VEGF) is a likely regulator of angiogenesis in the peripheral and central vascular systems. In the presence of VEGF, angiogenesis occurs, but in the absence of VEGF, the capillaries undergo apoptotic regression [35]. There appears to be an age-related decline in the capacity for cerebral angiogenesis that is associated with a reduction in VEGF expression [36]. A number of investigators have reported the downregulation of VEGF in brain tissues, which coincides with a reduction in brain capillary density. In the present study, isolated brain microvessels were used to examine the expression of proteins that mediate angiogenesis and accurately corresponded to the observed alterations in the brain microvessels; significantly lower VEGF expression was observed in the aging rats.

*In vitro* and* in vivo* experiments have demonstrated that eNOS enhances endothelial cell migration, proliferation, and differentiation [37–39]. It has been shown that blocking eNOS reduces VEGF-induced cell proliferation and migration [40]. The study of Reed and colleagues reported that defects in the activation of eNOS result in a decrease in NO production and contribute to impaired angiogenesis in aging [39]. In the brain vasculature, eNOS has been recently reported to play a role in maintaining preexisting collateral density in adulthood [41]. The present study also confirmed that brain microvascular eNOS expression was significantly diminished in the aging rats when compared with their young counterparts (Figure 8).

The angiogenic action of VEGF is mainly mediated by VEGF receptor 2 (KDR/Flk-1), which is primarily expressed by endothelial cells. The activation of the tyrosine kinase receptor Flk-1 in angiogenesis has been shown to be associated with the activation of eNOS in endothelial cells [26]. In aging, the impairment of Flk-1 has been demonstrated to result in the reduction of NO-mediated vasodilation in coronary arterioles [42]. Our data (Figure 7) demonstrated that the expression of brain microvessel Flk-1 was significantly lower in aging rats (although it was only significantly different in the immersed aged rats, a reduced trend was observed even in the sedentary-aged rats) when compared to young rats. This result is similar to Sun's group, which reported a reduction in Flk-1 expression in the cerebral vessels with advancing age using a fluorescence immunostaining technique [43]. They also demonstrated that the distribution of Flk-1 is found more in neurons than in vessels. This corresponds to our finding that a small amount of Flk-1 was present in the isolated brain microvessels of all of the animal groups. The reduced expression of Flk-1 in brain microvessels reflects the possibility that the other VEGF receptors, including VEGF receptor 1 (Flt-1), may be involved in the regulation of angiogenesis.

A large cross-sectional study provided evidence in humans that the resting blood flow velocity in the middle cerebral artery is elevated by habitual exercise across different ages [44]. In the present study, it was also determined that old rats showed significantly decreased rCBF when compared to young rats. Interestingly, our results showed that the training exercise program used in the present study could significantly increase the cerebral blood flow. This finding is in agreement with a previously published finding that the increased rCBF associated with training is mediated, at least in part, by local changes within the vasculature [44]. It has been reported that physical exercise ameliorates the age-induced impairment of angiogenesis and VEGF levels in several tissues [10, 12, 45].

Nitric oxide, which is produced by endothelial cells, is a regulating factor involved in CBF regulation [46]. It was found that CBF attenuation occurs in parallel with the downregulation of eNOS expression in spontaneously hypertensive rats [47]. Moreover, fluid shifts and arterial blood pressure elevation are associated with diminished middle cerebral artery eNOS protein levels, lower CBF, and higher cerebral vascular resistance [48]. Physical training has been shown to improve endothelium-dependent vasodilation, in part via the upregulation and increased phosphorylation of eNOS [49]. In old animals, exercise training ameliorates the reduction in endothelium-dependent vasodilation in muscle arterioles with decreased eNOS mRNA and protein levels [50]. Exercise training restores the age-related decrease in flow-induced vasodilatation in the muscle arterioles [51]. Using L-NAME, the inhibition of vasodilator responses was found to be increased after exercise training, suggesting that the exercise training-induced enhancement of flow-induced dilation occurs primarily through an NO-related mechanism [52]. The present study is the first to demonstrate that exercise training is capable of markedly enhancing eNOS expression in the brain microvessels of aging rats.

Although the mechanism by which regular physical activity induces beneficial effects in the cerebral vasculature has not been elucidated, it is possible that the repeated exposure to increases in blood flow and shear stress in specific regions of the brain during exercise play a role [53]. The previous study demonstrated that cerebral vessels in exercised animals have a smooth surface, which may facilitate laminar blood flow and make them less prone to thrombogenic events than sedentary vessels [51]. Indeed, there is consistent evidence that blood flow is increased in some areas of the brain during physical activity [54]. The increase in cerebral blood flow seems to be linked to the local vasodilator action of metabolites and/or other local effects produced by increased neural activity during exercise [55]. A large body of literature suggests that shear stress may also be a signal for endothelial adaptations, particularly in the vasculatures of noncontracting tissues. The concept that shear stress can signal the endothelial function to alter is well supported by* in vitro* [56] and* in vivo* data [57]. Vascular bed flow-induced dilation is known to be mediated predominantly by the production of NO from eNOS [58]. For the cerebral vasculature, it has been reported that flow-induced vasodilation* in vivo* involves the activation of NOS and the generation of NO [59]. Recently, the D'Amore group demonstrated that cultured human umbilical vein endothelial cells (HUVECs) exposed to shear stress show an increase in VEGF and Flk-1 expression compared with a static control [60]. Fluid shear stress also regulates endothelial sprouting in a NO-dependent manner* in vitro* [13]. Moreover, an* in vivo* study determined that increased capillary shear stress induces angiogenesis [14].

It has been well established that mitochondrial DNA accumulates mutations with aging [61]. A decrease in the mitochondrial antioxidant superoxide dismutase (MnSOD) has also been found in the cerebrovasculature with increasing age [62]. The exposure of brain endothelial cells to oxidized lipids increases NO and ROS production [63]. Approximately 90% of cellular ROS production is attributable to the mitochondria [61]. Although the present study did not determine the lipid peroxide level in either the brain tissue or isolated microvessels, the plasma MDA level is commonly used as an indicator of oxidative damage. Our data demonstrates the relationship between brain capillary vascularity and the plasma MDA level in all of the animal groups. The significant negative correlation between the CV and MDA level shown in Figure 9 indicates that exercise training may also improve age-induced brain microvascular deterioration via an antioxidant mechanism.

## 5. Conclusion

Our findings indicate that the effective mechanisms of exercise training on age-induced brain microvascular changes involved the upregulation of VEGF and eNOS expression in association with changes in the oxidant-antioxidant balance.

## Figures and Tables

**Table 1:** Body weight (g), mean arterial blood pressure (mmHg), systolic blood pressure (mmHg), diastolic blood pressure (mmHg), and plasma malondialdehyde (nmol/mL) in theSE-Young, SE-Aged, IM-Aged, and ET-Aged groups.

	SE-Young	SE-Aged	IM-Aged	ET-Aged
Body weight (g)	474.80 ± 10.17 (5)	675.50 ± 19.36* (8)	686.80 ± 16.87* (5)	591.50 ± 14.68^∗,#^ (8)
Mean arterial blood pressure (mmHg)	99.11 ± 6.04 (5)	130.00 ± 5.00* (8)	130.39 ± 2.69* (5)	115.79 ± 4.30^∗,#^ (7)
Systolic blood pressure (mmHg)	121.00 ± 5.95 (5)	144.58 ± 5.07* (8)	144.83 ± 3.43* (5)	130.42 ± 4.10 (7)
Diastolic blood pressure (mmHg)	88.17 ± 6.41 (5)	122.71 ± 5.03* (8)	123.176 ± 2.64* (5)	106.90 ± 4.48^∗,#^ (7)
Plasma malondialdehyde (nmol/mL)	1.84 ± 0.46 (5)	11.93 ± 2.28* (6)	12.08 ± 0.94* (5)	5.32 ± 1.60^#^ (5)

Values are expressed as the mean ± SEM, and the number of rats is shown in parentheses.

*P < 0.05, significantly different from the SE-Young group.

^#^ *P* < 0.05, significantly different from the IM-Aged group.
